# Functional Characterization of a Prokaryotic Kir Channel

**DOI:** 10.1074/jbc.C400417200

**Published:** 2004-09-23

**Authors:** Decha Enkvetchakul, Jaya Bhattacharyya, Iana Jeliazkova, Darcy K. Groesbeck, Catherine A. Cukras, Colin G. Nichols

**Affiliations:** ¶ Department of Cell Biology and Physiology, Washington University School of Medicine, St. Louis, Missouri 63110; ‡ Division of Renal Medicine, Washington University School of Medicine, St. Louis, Missouri 63110

## Abstract

The Kir gene family encodes inward rectifying K^+^ (Kir) channels that are widespread and critical regulators of excitability in eukaryotic cells. A related gene family (KirBac) has recently been identified in prokaryotes. While a crystal structure of one member, KirBac1.1, has been solved, there has been no functional characterization of any KirBac gene products. Here we present functional characterization of KirBac1.1 reconstituted in liposomes. Utilizing a ^86^Rb^+^ uptake assay, we demonstrate that KirBac1.1 generates a K^+^-selective permeation path that is inhibited by extraliposomal Ba^2+^ and Ca^2+^ ions. In contrast to KcsA (an acid-activated bacterial potassium channel), KirBac1.1 is inhibited by extraliposomal acid (p*K_a_* ~ 6). This characterization of KirBac1.1 activity now paves the way for further correlation of structure and function in this model Kir channel.

Inwardly rectifying potassium channel (KCNJ) genes encode a large and widespread eukaryotic Kir channel family that modulates excitability throughout the organism (1). A potentially equally large family of prokaryotic genes encoding putative bacterial Kir channels (KirBacs) was found in bacterial genomes (2). One member, KirBac1.1, was successfully crystallized (3), and its structure is consistent with a potassium channel (4, 5). Surprisingly, despite this detailed structural insight, there has been no functional characterization of any KirBac proteins and no evidence that KirBac1.1 functions as a channel. Potassium channel properties of KcsA (6) were originally characterized using a ^86^Rb^+^ uptake assay of reconstituted purified proteins in liposomes (7-9), an assay with the advantage of examining the ensemble behavior of many channels. We cloned full-length KirBac1.1, purified it in milligram quantities, and used a similar liposome assay to study its function. Purified KirBac1.1 reconstituted in liposomes indeed forms a K^+^-selective conductance (K^+^~ Rb^+^ ~Cs^+^ ≫ Li^+^~ Na^+^~ NMGM^+^)^1^ that is inhibited by Ba^2+^ and Ca^2+^. In contrast to KcsA, but consistent with eukaryotic Kir channels, KirBac1.1 is inhibited at low pH. This characterization of KirBac1.1 activity now paves the way for further correlation of structure and function in this model Kir channel.

## MATERIALS AND METHODS

### Molecular Biology—

**Fig. 1.**
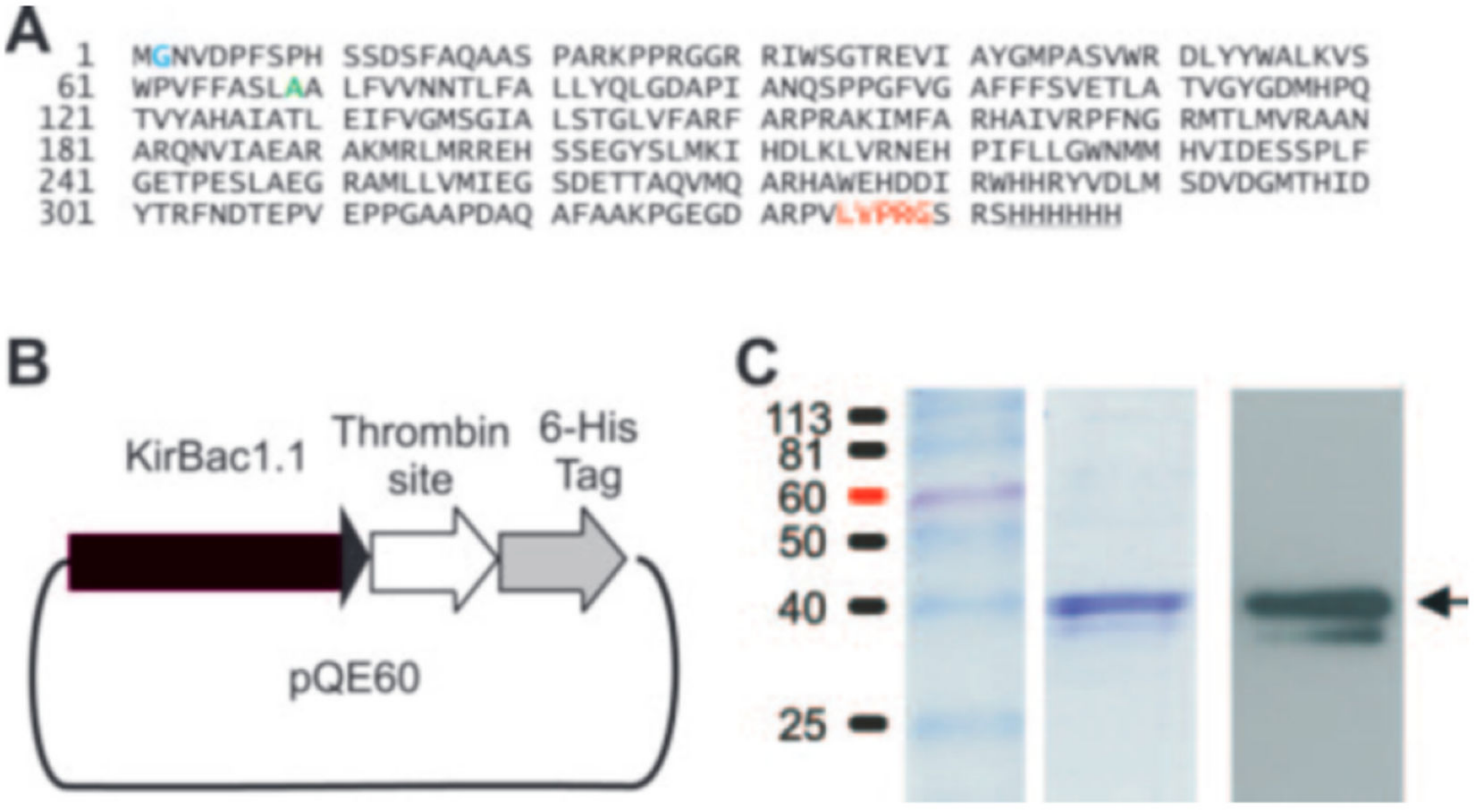
Expression of KirBac1.1. *A*, sequence of KirBac1.1. Inserted Gly at position 2 (*blue*), Ala at position 69 (mutated to valine in the crystallized channel (Kuo *et al.*, 2003), *green*), C-terminal thrombin cleavage sequence (*red*), and His_6_ tag (*underlined*) are indicated. *B*, schematic of KirBac1.1 expression construct. Full-length KirBac1.1 was expressed as C-terminal His_6_-tagged protein with intervening thrombin site and affinity-purified on a cobalt column (see “Materials and Methods”). *C*, purified protein was run on SDS-PAGE and stained with Coomassie Brilliant Blue (*middle lane*) or transferred to nitrocellulose and probed with anti-6-histidine antibodies (*right lane*). The standardized protein ladder is shown in the *left lane*.

KirBac1.1 was cloned from genomic DNA of *Burkholderia pseudomallei* by PCR. For expression in *Escherichia coli*, an NcoI restriction site was attached at the 5′ end of KirBac1.1 and a segment of DNA coding for the amino acids “LVPR” followed by a BamHI site replaced the stop codon at the 3′ end by PCR. The product was subcloned into NcoI and BamHI sites of the pQE60 vector (Qiagen Inc.) resulting in a fusion construct coding for KirBac1.1, immediately followed by the amino acid sequence “LVPRGSRSHHHHHH” that contains a thrombin cleavage site and a His_6_ tag used for purification (Fig. 1, A and B). An extra glycine was introduced after the initiator methionine to keep the sequence in frame.

### Protein Purification—

Transformed BL21*(DE3) were grown in 1-liter cultures to *A*_600_ ~1.0, induced with 0.5 mm isopropyl *β*-d-thiogalactopyranoside at 37C for 3 h, pelleted, and resuspended in 20 ml of 50 mm Tris-HCl, pH 8.0, 150 mm KCl, and two tablets of EDTA-free protease inhibitor mixture tablets (Roche Diagnostics). Bacteria were lysed by sonication; decylmaltoside (Anatrace) was added to a final concentration of 30 mm and incubated at room temperature for 2–4 h. The mixture was centrifuged at 30,000 × *g* for 30 min, and the supernatant was applied to a cobalt affinity column. The column was washed with 20–30 bed volumes of wash buffer (50 mm Tris-HCl, pH 8.0, 150 mm KCl, 10 mm imidazole, and 5 mm decylmaltoside) to minimize nonspecific binding. KirBac1.1 was eluted with 1–2 ml of wash buffer containing 500 mm imidazole. One liter of culture typically yielded ~1 mg of KirBac1.1 protein and was concentrated to 1–8 mg/ml using 30-kDa centrifugal filters (Millipore) and stored in wash buffer with 20 mm imidazole. The concentration was quantified by Pierce protein assay or optical densitometry using an estimated molar extinction coefficient of 51910 m^−1^ cm^−1^ (10).

### ^86^Rb^+^ Uptake Assay—

Composition of buffers is as follows: buffer A, 450 mm KCl, 10 mm HEPES, 4 mm NMG, pH 7; buffer B, 450 mm sorbitol, 10 mm HEPES, 4 mm NMG, 50 *μ*m KCl, pH 7.0. Lipids at 3:1 POPE:POPG (Avanti) were dried under nitrogen followed by vacuum centrifugation and stored in 4-mg aliquots at −4 °C in N_2_-filled glass tubes covered with parafilm. Lipid aliquots were resuspended (10 mg/ml in buffer A) using a bath sonicator, CHAPS was added to a final concentration of 37 mm, and the mixture was incubated at room temperature for 2 h before assay. Purified KirBac1.1 (or KcsA) was added (2–3 *μ*g/ml lipid, volume of protein ~0.5–3 *μ*l per 100 *μ*l of lipids) and incubated 30 min. Disposable polystyrene columns (Pierce, catalog number 29920) were packed with Sephadex G-50 (fine) beads (1 ml), swollen overnight in buffer A or B. Prespun columns (filled with Sephadex beads swollen in buffers A and B, respectively) were prepared immediately prior to use by centrifugation at 1500 × *g* (Beckman TJ6); centrifugation was stopped upon reaching the goal speed. Liposomes were formed by placing 100 *μ*l of detergent-solubilized lipid/protein mixture directly on top of the gel bed of a prespun column A, and liposomes were collected into glass tubes by spinning at 1000 × *g* (2500 RPM Beckman TJ6) and immediately stopping upon reaching goal speed. Liposomes were used within 0.5–4 h. Immediately before flux measurements, extraliposomal solution was exchanged for buffer B by placing the suspension directly on top of the gel bed of a prespun column B, and liposomes were collected by centrifugation at 1000 × *g*. ^86^Rb^+^ uptake was initiated by mixing liposomes with 4 volumes buffer B (final concentration of ^86^Rb^+^ at 1–5 *μ*m). A 50-*μ*l aliquot of the radioactive mixture was taken at the time points indicated, and extraliposomal ^86^Rb^+^ was removed by passage over a 0.5-ml Dowex cation exchange column in the NMGH^+^ form and eluting into scintillation vials with 1.5 ml of 400 mm sorbitol. Samples were mixed with scintillation fluid and counted in a liquid scintillation counter. Dowex beads were washed in methanol and distilled water and converted to NMGH^+^ form by addition of HCl then NMG. Disposable polypropylene screening columns (Fisher, catalog number 11-387-50) were filled with 0.5 bed volume of the Dowex beads. Dowex columns were further prepared by applying 1 ml of bovine serum albumin (5 mg/ml) in 400 mm sorbitol to minimize nonspecific binding and 50 *μ*l of liposome solution to minimize reduced recovery of liposomes that tended to occur with the first set of samples, followed by 2 ml of 400 mm sorbitol. Valinomycin was used to assay maximal ^86^Rb^+^ uptake.

## RESULTS

### Cloning of Full-length KirBac1.1—

We cloned KirBac1.1 (11) from genomic *B. pseudomallei* DNA (from bacterial lysate, a gift of Richard Titball and Tim Atkins, Defense Science and Technology Laboratory, Porton Down, Salisbury, Wiltshire, UK). The cloned sequence (see Fig. 1A) is identical to that published in the data base (*B. pseudomallei* Sequencing Group, ftp.sanger.ac.uk/pub/pathogens/bps/) and differs from the protein crystallized by Kuo *et al.* (3), which contained a point substitution (A69V) and a 12-amino acid deletion from the C terminus).

### ^86^Rb^+^ Uptake Liposomal Assay of Purified KirBac1.1 Protein—

**Fig. 2.**
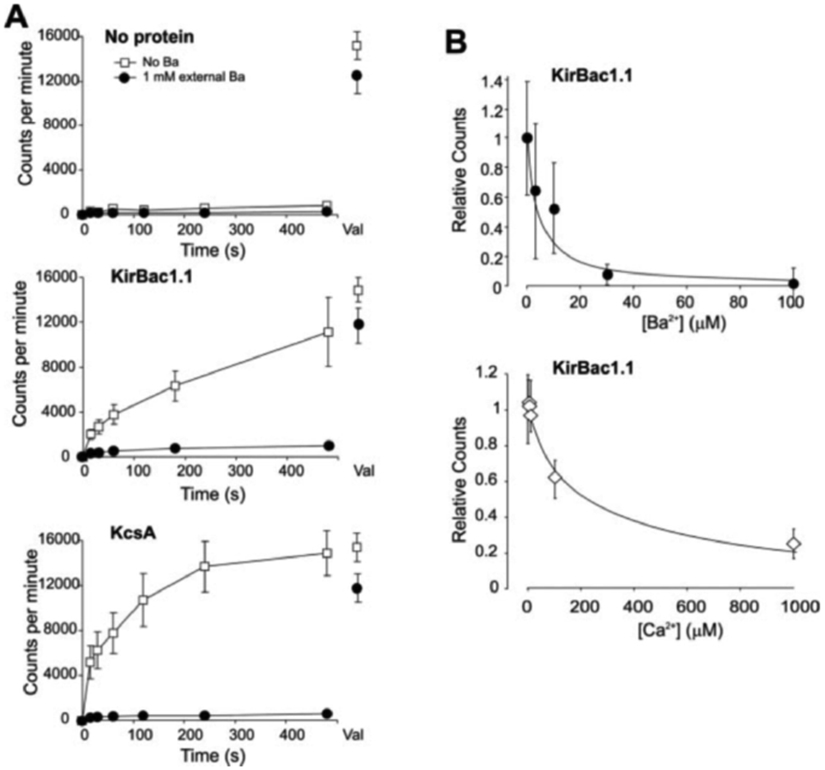
Functional assay of reconstituted KirBac1.1. *A*, time course of ^86^Rb^+^ uptake into liposomes (POPE:POPG, 75: 25%), with 450 mm internal and 0.05 mm external [K^+^]. Liposomes were reconstituted with no protein, KirBac1.1 (2.5 *μ*g/mg lipid), or KcsA (2 *μ*g/mg lipid). Experiments were performed with (*filled circles*) or without (*open squares*) 1 mm extraliposomal Ba^2+^. Uptake following addition of valinomycin (*Val*) to the vesicles is indicated to the *right*. *B*, ^86^Rb^+^ uptake in liposomes reconstituted with KirBac1.1 as a function of external [Ba^2+^] or [Ca^2+^] (relative to zero divalents) measured at 30 s, *n* = 3–5, *error bars* = S.E. Fits of the Hill equation are shown, with *H* = 1, *K*_½_ = 4 *μ*m (Ba^2+^) and *H* = 0.9, *K*_½_ = 220 *μ*m (Ca^2+^).

C-terminal His_6_-tagged KirBac1.1 (Fig. 1, A and B) was expressed in *E. coli* and purified over a cobalt column (see “Materials and Methods”). By Coomassie Blue stain and Western blot (Fig. 1C), purified KirBac1.1 migrates as a single major band (~40 kDa), with a faint band at a slightly lower molecular mass on SDS-PAGE, consistent with the expected monomeric molecular mass (38.9 kDa including thrombin site and His_6_ tag). Purified KirBac1.1 was reconstituted into liposomes (POPE:POPG 3:1 ratio) formed with 450 and 0.05 mm internal and external [K^+^], respectively (see “Materials and Methods”). Liposomes that contain a K^+^-selective permeation pathway will generate a large negative internal potential due to this large K^+^ gradient that will drive uptake of tracer ^86^Rb^+^ added to the external solution, allowing uptake of ^86^Rb^+^ to be used as a surrogate for K^+^-selective channel activity. Fig. 2A shows ^86^Rb^+^ uptake into liposomes reconstituted with no protein, KirBac1.1, or KcsA, at neutral pH. Liposomes without protein demonstrate very low uptake rates (Fig. 2A). As a positive control, KcsA forms a K^+^-selective pathway when reconstituted in liposomes (8, 9) and shows rapid, Ba^2+^-inhibitable uptake (Fig. 2A). KirBac1.1 also demonstrates a robust ^86^Rb^+^ uptake, indicating that KirBac1.1 protein produces a K^+^ permeation pathway, selective for K^+^ over Cl^−^.

KirBac1.1 is also markedly inhibited by externally applied 1 mm Ba^2+^ and Ca^2+^ (Fig. 2). Barium and Ca^2+^ sensitivity were assessed by Rb^+^ uptake at 30 s (*i.e.* before saturation), as a fraction of the uptake in the absence of divalents. KirBac1.1 inhibition by Ba^2+^ and Ca^2+^ are concentration-dependent, with half-maximal inhibition at ~4 and ~220 *μ*m, respectively (Fig. 2B).

### KirBac1.1 Is Selective for K^+^ over Na^+^—

**Fig. 3.**
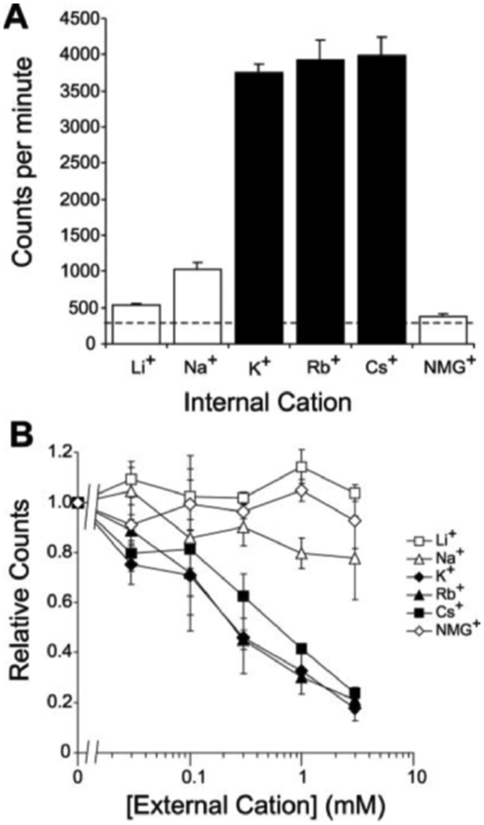
Cation selectivity of KirBac1.1. *A*, liposomes with reconstituted KirBac1.1 (2.5 *μ*g/mg lipid) were formed with 450 mm indicated cation and 0.05 mm external [K^+^] at pH 7.4. ^86^Rb^+^ uptake was measured at 8 min. The *dashed line* indicates background uptake in K^+^ gradient, in the absence of protein. *B*, liposomes were made with 450 mm internal and 0.05 mm external [K^+^]. Additional cations were added externally at the concentrations indicated. ^86^Rb^+^ uptake was measured at 8 min and normalized to counts with zero added cations. All measurements were made in triplicate (mean ± S.E.).

Potassium channels are highly selective for K^+^, Rb^+^, and Cs^+^ over other alkali metal ions (12). In liposomes reconstituted with a cation permeation pathway, substitution of the internal permeant cation with a non-permeant cation would prevent development of negative internal liposomal potential and diminish ^86^Rb^+^ uptake. Using this strategy, we replaced internal K^+^ with Li^+^, Na^+^, Rb^+^, Cs^+^, or NMGH^+^ ions to assess cation selectivity of KirBac1.1 (Fig. 3A). Liposomes containing KirBac1.1, with K^+^, Rb^+^, or Cs^+^ as the internal cation, demonstrate significant uptake, consistent with these ions being permeant. Substitution with Na^+^, Li^+^, or NMGH^+^ resulted in minimal uptake of ^86^Rb^+^, indicating that these cations are not significantly permeant.

The strong negative internal potential in liposomes is created secondarily to the large concentration gradient of permeant cations. The addition of permeant cations to the external solution will diminish this electrical gradient and decrease ^86^Rb^+^ uptake, allowing an alternative method of assessing cation selectivity. Liposomes with reconstituted KirBac1.1 were formed with 450 mm internal K^+^, 0.05 mm external K^+^, and varying concentrations of Li^+^, Na^+^, K^+^, Rb^+^, Cs^+^, or NMGH^+^ ions were added to the external solution (Fig. 3B). Increasing external K^+^, Rb^+^, and to a slightly lesser extent Cs^+^, results in a decreased uptake of ^86^Rb^+^, again consistent with these ions, but not Li^+^, Na^+^ NMGH^+^, being permeant.

These results show that KirBac1.1 forms a cation pathway with selectivity for K^+^, Rb^+^, and Cs^+^ over Li^+^, Na^+^, and NMGH^+^, blocked by divalent Ba^2+^ and Ca^2+^. Polyamines are the characteristic pore blockers of strongly rectifying eukaryotic Kir channels (1). We attempted to examine blocking of KcsA- and KirBac1.1-mediated ^86^Rb^+^ uptake by extraliposomally applied polyamines. Below 100 mm, there was no evidence of channel block by spermidine or spermine. At higher concentrations, valinomycin-dependent uptake is abolished, suggesting that these polycations collapse the electrochemical gradient, probably by destroying the liposomes (data not shown). We also tested toxin blockers of K^+^ channels, TertiapinQ (TPNQ), and charybdotoxin (CTX), which act through binding to the external side of the channel pore (13-15). TPNQ selectively blocks two members of the Kir family, Kir3.1/3.4 and Kir1.1, but not Kir6; CTX blocks certain voltage- and calcium-activated potassium channels. In liposomes reconstituted with KirBac1.1 or KcsA, up to 1 mm extraliposomal and 10 *μ*m intraliposomal TPNQ and CTX both failed to inhibit uptake (data not shown).

All eukaryotic Kir channels are activated by the addition of phospholipids, particularly phosphatidylinositol 4,5-bisphosphate (PIP_2_) to the cytoplasmic face of the membrane and it is believed that PIP_2_ or other negatively charged lipids are required for channel activity (16). PIP_2_ is generally absent from prokaryotic membranes and obviously not necessary for activity of KirBac1.1 in pure POPE:POPG membranes, but experiments to examine the lipid requirements for channel activity are clearly warranted. Finally, ATP can activate many Kir channels by phosphorylation and specifically inhibits Kir6 subfamily channels by binding to the cytoplasmic domain of the channel (16). Addition of ATP at up to 10 mm (Na^+^ salt) was without effect on KirBac1.1 (data not shown).

### pH Modulation of KirBac1.1 Activity—

**Fig. 4.**
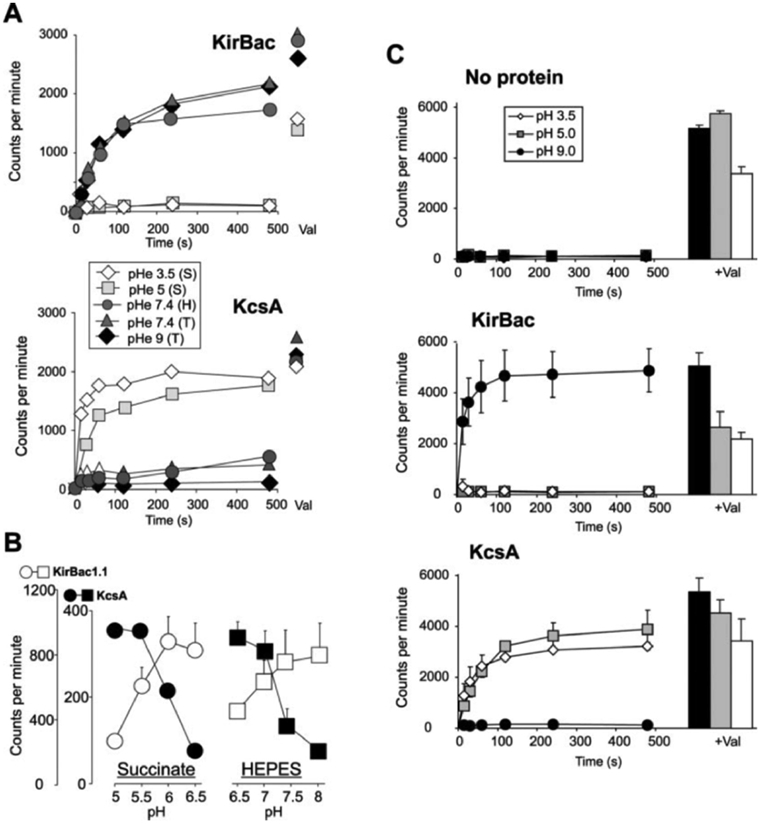
pH dependence of KirBac1.1 activity. *A*, time course of ^86^Rb^+^ uptake in liposomes reconstituted with KirBac1.1 or KcsA. External pH (pH_e_) was adjusted to 3.5–9 using succinate (*S*), HEPES (*H*), or Tris (*T*) buffers as indicated. Internal pH was originally buffered to pH 7.0 with HEPES. *B*, ^86^Rb^+^ uptake at 8 min as a function of pH_e_ for KcsA (*filled, symbols*) and KirBac1.1 (*open symbols*), using succinate (*left*)- or HEPES (*right*)-buffered solutions. Internal liposomal pH was 7.0 with HEPES throughout (*n* = 3, mean ± S.E.). *C*, time course of ^86^Rb^+^ uptake in liposomes reconstituted with no protein, KirBac1.1, or KcsA. pH_e_ was adjusted to 3.5, 5 (using succinate), or 9 (using Tris. Internal pH was originally buffered to 7.0 with HEPES. To the *right* are mean (+S.E.) uptakes after addition of valinomycin.

Protons modulate eukaryotic Kir activity, and Kir1, Kir4, and Kir5 are particularly sensitive to inhibition by intracellular protons (17). We examined the pH dependence of ^86^Rb^+^ uptake by KirBac1.1 in liposomes formed with an internal pH of 7.0 and then placed in varying external pH. As demonstrated previously (9), the time course of ^86^Rb^+^ uptake was markedly inhibited by high external pH in liposomes reconstituted with KcsA (Fig. 4A). In contrast, KirBac1.1 demonstrated an opposite dependence on external pH and was completely inhibited at pH 5 and below (Fig. 4B). To achieve the indicated pH levels, three different external buffers were used, succinate (pH 3.5 and 5), HEPES (pH 7.4), and Tris (pH 7.4 and 9). Conceivably, the difference in activation of KirBac and KcsA could be due to the different buffers themselves, rather than proton concentration. We therefore examined the pH dependence of ^86^Rb^+^ uptake over narrower ranges using single buffer systems (Fig. 4B, *left* (Succinate) and right (*HEPES*)). While this precludes examination of a wide pH range, it is clear that there is activation of KcsA, but inhibition of KirBac1.1, below neutral pH. Unexpectedly, the activity curve for both KcsA and KirBac1.1 is shifted ~1.5 pH units more alkaline in HEPES-buffered solutions compared with succinate-buffered solutions. The cause of the shift is unclear; pH of the solutions was confirmed using both a pH electrode and fluorometrically (phenol red), before and after the experiment to ensure stability.

In the representative experiments of Fig. 4A, it is apparent that in KirBac1.1-containing liposomes there is a reduced valinomycin flux as pH is lowered below neutral. We examined this systematically using different protein preparations (Fig. 4C). Although more pronounced for KirBac1.1, it is apparent that both KirBac1.1 and KcsA are associated with a reduced valinomycin uptake, as pH is made more acidic. At pH 3.5, valinomycin flux is significantly reduced even in liposomes without protein, reflecting an additional non-protein component.

## DISCUSSION

To date, three prokaryotic potassium channels have been functionally characterized, KcsA, MthK and KvAP (6-9, 17, 18). Two of these (MthK and KvAP) represent ancestral homologs of eukaryotic Ca-activated (KCa) and voltage-gated (Kv) potassium channels, respectively. The available crystal structures provide a template for modeling and understanding the molecular basis of KCa and Kv channel activity. KcsA is something of an anomaly, being an acid-activated two-transmembrane helix channel without obvious eukaryotic descendents. The eukaryotic Kir (KCNJ1–7) gene family expresses Kir channels with critical roles in most cell types (1). The defining structural feature of Kir channels is a conserved cytoplasmic domain (5) that provides for a rich and varied regulation of these channels by intracellular ligands (16, 19, 20). A prokaryotic Kir channel family has now appeared in the data base (2), and the crystallization of one member (3) provides a critical high-resolution structure of an archetypal Kir channel (KirBac1.1). Uniquely, KirBac1.1 is the only potassium channel protein to have been crystallized (3) without any information regarding its function. The present data demonstrate that full-length KirBac1.1 does generate a potassium-selective permeation path in phospholipid membranes, with important regulatory features that are consistent with those of eukaryotic Kir channels.

### Ion Transport Properties of KirBac1.1—

Both KirBac1.1 and KcsA generate permeability to K^+^ and the close analogues Rb^+^ and Cs^+^. Na^+^, Li^+^, and organic cations are essentially impermeable, and submillimolar Ca^2+^ or Ba^2+^ blocks both channels (8). The data indicate similar permeability to K^+^, Rb^+^, and Cs^+^ in KirBac1.1. In mammalian Kir channels, K^+^ is generally more permeant than Rb^+^ or Cs^+^. Residues Thr-141 and Ser-165 in Kir2.1 both contribute to conductance and block by Cs^+^ and Rb^+^ ions (21). Mutation of these two residues to alanine and leucine, respectively, leads to increased Rb^+^ permeation and large Cs^+^ permeation. Interestingly, the corresponding residues in KirBac1.1 are alanine and isoleucine (at positions 110 and 132 in Fig. 1A), which may explain the high Rb^+^ and Cs^+^ permeability indicated by the ion substitution experiments in Fig. 3. Further experiments exploring the role of these residues in controlling ion selectivity are warranted.

### pH Dependence of KirBac1.1—

KcsA is activated under acidic extraliposomal conditions (Fig. 4), consistent with published studies (9, 13). In marked contrast, KirBac1.1 activity is inhibited in extraliposomal acidic conditions. In an attempt to examine intraliposomal pH dependence of activity, we formed liposomes in different pH and then washed them in external pH 7.0. There was no obvious dependence of either KirBac or KcsA activity on intraliposomal pH (data not shown). This suggests that neither KcsA nor KirBac1.1 are sensitive to intraliposomal pH. One possible interpretation is that both KcsA and KirBac1.1 are oriented unidirectionally with the pH sensor pointed extraliposomally. Alternatively, channels could be oriented bidirectionally and function only if the pH sensor is directed outward. However, an unavoidable possibility is that the pH gradient across the liposome will equilibrate to the extraliposomal pH, which thus modulates channels in either orientation.

The cause of the differential effect of succinate *versus* HEPES buffer on KcsA and KirBac1.1 is unclear, but since both channels show similar parallel (~1.5 pH units) shifts of activation curves, it seems likely that there is a systematic buffer effect on intraliposomal, extraliposomal, or intramembrane pH. Conceivably, diffusion of uncharged buffer into liposomes could cause acidification by succinic acid or uncharged HEPES. If functional channels are oriented with their pH sensors inwards, a differential diffusion could cause the observed shift in apparent extraliposomal pH dependence. The differential effect of buffer system could also result from a direct effect itself on the channels (22). Any direct effect of the buffer would have to be the same on both KcsA and KirBac1.1 and would not affect the conclusion that KirBac1.1 is inhibited by extraliposomal protons, opposite to the pH sensitivity of KcsA. Inhibition by low cytoplasmic pH is a common feature of eukaryotic Kirs. Of the latter, Kir1.1 and Kir4.1/Kir5.1 channels are particularly sensitive to proton inhibition (16, 23, 24). While the mechanism of proton inhibition in eukaryotic Kir channels is still not entirely clear, it seems likely that common pH-sensing elements (24-26) are present in the cytoplasmic domain of both eukaryotic and prokaryotic channels.

### Conclusions—

KirBac1.1 has been crystallized, but there has been no functional characterization of any prokaryotic Kir proteins. The above data demonstrate that purified KirBac1.1 forms a potassium-selective permeation pathway when reconstituted into liposomes, with typical K^+^ channel ion selectivity as well as sensitivity to block by Ba^2+^ and Ca^2+^. In direct contrast to KcsA, but consistent with eukaryotic Kir channels, KirBac1.1 is inhibited in acidic conditions. The combination of functional characterization of mutant KirBac1.1 and crystallographic studies (3) can now be used to take understanding of the mechanisms of activity and regulation of Kir channels to a new level.

## Footnotes

1 The abbreviations used are: NMGM^+^, protonated NMG; NMG, *N*-methyl-d-glucamine; POPE, palmitoyl oleoyl phosphatidylethanolamine; POPG, phosphatidylglycerol; CHAPS, 3-[(3-cholamidopropyl)dimethylammonio]-1-propanesulfonic acid; TPNQ, TertiapinQ; CTX, charybdotoxin; PIP_2_, phosphatidylinositol 4,5-bisphosphate.
